# Long‐Term Outcomes After Slowly Resorbable P4HB Mesh Implantation: A Multicenter Analysis From European Registry

**DOI:** 10.1002/wjs.70331

**Published:** 2026-03-20

**Authors:** Rudolf van den Berg, Marie Wieser, Manuel López‐Cano, José Bueno‐Lledó, Ferdinand Köckerling, Grigorios Chatzimavroudis, Constanza Gonella‐Pacchiotti, Cesare Stabilini, Pablo Ortega‐Deballon, Benoit Romain, PRS Group (Phasix Research Study Group), Giorgio Soliani, Lars N. Jorgensen, Jose Antonio Pereira Rodriguez, Jean‐François Gillion, Vincent Dubuisson, Niki Christou, Yohann Renard, David Moszkowicz, Guillaume Péré, Gregory Baud, Guillaume Passot, Benjamin Barrat, J. Jeekel

**Affiliations:** ^1^ Department of Surgery Erasmus University Medical Centre Rotterdam the Netherlands; ^2^ Department of Digestive Surgery University of Strasbourg, Hautepierre Hospital Strasbourg France; ^3^ Abdominal Wall Surgery Unit, Department of General Surgery Hospital Universitari Vall d'Hebron, Unviversitat Autònoma de Barcelona Barcelona Spain; ^4^ Unit of Abdominal Wall Surgery, Department of Digestive Surgery Hospital Universitari I Poltecnic La Fe, Fernando Abril Martorell Valencia Spain; ^5^ Hernia Center Vivantes Humboldt‐Hospital, Academic Teaching Hospital of Charité University Medicine Berlin Germany; ^6^ Complex Hernia & Abdominal Wall Reconstruction Center European Interbalkan Medical Center Thessaloniki Greece; ^7^ Department of Surgery S. Anna University Hospital and University of Ferrara Ferrara Italy; ^8^ Department of Surgery, Policlinico San Martino IRCCS and Department of Surgical Sciences University of Genoa Genoa Italy; ^9^ Department of Digestive, Thoracic and Surgical Oncology University Hospital Dijon France

**Keywords:** Phasix mesh, postoperative complications, resorbable mesh, ventral hernia

## Abstract

**Background:**

Fully resorbable biosynthetic mesh composed of poly‐4‐hydroxybutyrate (P4HB), have been designed for incisional hernia (IH) repair, including in contaminated surgical fields. While existing studies have demonstrated its safety and efficacy in the short term, comprehensive long‐term data, particularly after complete mesh resorption, remain scarce.

**Methods:**

This multicenter European registry analysis investigates the incidence of short‐ and long‐term complications following IH repair with Phasix (P4HB) or Phasix ST (P4HB with hydrogel barrier; BD, Warwick, RI, USA) mesh. Adult patients from registries in France, Greece, Germany, and Spain were included and stratified using the Ventral Hernia Working Group (VHWG) classification. The primary endpoint was the incidence of long‐term mesh‐related complications one to five years post‐implantation, after mesh resorption. Secondary endpoints included short‐term complications and hernia recurrence.

**Results:**

A total of 790 patients underwent incisional hernia repair with P4HB mesh, with a median follow‐up of 38 months (IQR 36–48). Long‐term follow‐up beyond 24 months was available in 57% of patients. The majority of complications occur during the first 6 months. Long‐term mesh‐related morbidity remained low, with mesh infection occurring in 2% of patients and chronic pain in 3%–5%, even after complete mesh resorption. Rates of enterocutaneous fistula and mesh explantation were rare. Long‐term complication profiles varied by hernia complexity, comorbidity burden, and mesh position, with higher ASA class associated with increased risk of mesh infection and chronic pain. The overall hernia recurrence rate was 22%, with recurrence increasing after the expected resorption period and stabilizing thereafter. Higher recurrence risk was independently associated with VHWG grade III–IV (HR of 2.55 and 2.49), obesity (HR 1.41), and intraperitoneal mesh placement (HR 2.72).

**Conclusion:**

P4HB mesh demonstrated a favorable long‐term safety profile after complete resorption, with low rates of mesh‐related complications, even in high‐risk patients. Hernia recurrence remains an important secondary outcome and is strongly influenced by patient risk factors and surgical technique. These findings support a tailored, risk‐stratified approach to the use of biosynthetic meshes in IH repair.

## Graphical abstract

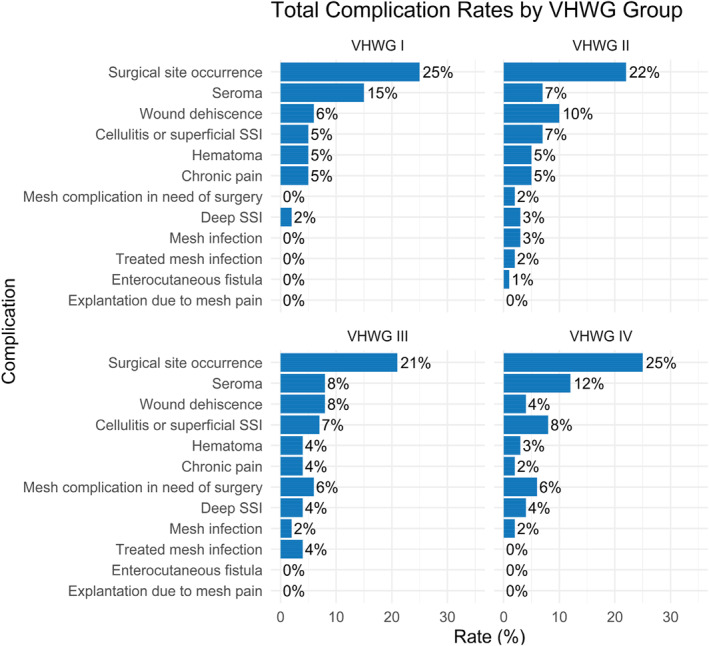

## 1 Introduction

The use of biosynthetic (i.e., fully resorbable) meshes in incisional hernia (IH) repair has gained increasing interest in recent years, particularly in complex or contaminated surgical fields where non‐resorbable synthetic materials may lead to higher rates of complications [1, 2, 3]. Poly‐4‐hydroxybutyrate (P4HB) mesh is a fully resorbable mesh designed to provide temporary reinforcement of the abdominal wall during the healing process [4]. The material is eliminated by the body in 12–18 months through hydrolytic enzymatic degradation after which tissue integrity must be maintained by the patient's own tissue regeneration [5, 6].

Several studies have demonstrated that P4HB mesh is safe and effective on the short term even in contaminated fields, with acceptable rates of surgical site occurrences (SSOs) and surgical site infections (SSIs) [5, 6, 7]. Long‐term recurrence rates appear to be comparable to those of non‐resorbable synthetic meshes [1, 6, 8]. Reported recurrence rates vary between 5.7% and 31.7%, depending on patient selection, hernia complexity, and duration of follow‐up [5, 6]. A recent meta‐analysis reported a pooled recurrence rate of 9% with a median follow‐up of over 5 years [2].

Despite these promising results, long‐term data regarding mesh‐related complications after complete resorption of the P4HB mesh remain scarce [9]. While complications such as chronic pain, fistula formation, and mesh infections are well‐documented with non‐resorbable meshes [10], few studies have addressed whether similar late‐onset adverse events occur with biosynthetic meshes after their complete degradation [6]. Additionally, concerns persist regarding the structural stability of the abdominal wall following mesh resorption, particularly in high‐risk patients with comorbidities or prior wound infections [11].

To address this knowledge gap, we conducted a large, multicenter European registry‐based study evaluating short‐ and long‐term complications following IH repair using P4HB or P4HB with hydrogel barrier mesh. The primary aim was to assess the incidence and nature of long‐term complications postoperatively, specifically after the expected resorption period of the mesh. Secondary objectives included the assessment of hernia recurrence and short‐term postoperative complications.

## 2 Methods

### 2.1 Study Design and Setting

We conducted an observational study using prospective data from several European registries in abdominal wall surgery. Participating centers were located in France, Germany, Denmark, Greece, Italy, Portugal, and Spain. Each center included consecutive patients who underwent incisional hernia repair (IHR) using either Phasix (P4HB; BD, Warwick, RI, USA) or Phasix ST (P4HB with hydrogel barrier; BD, Warwick, RI, USA) mesh. The protocol for this study was published in detail elsewhere [12].

P4HB mesh was generally placed in a retromuscular (sublay) or onlay position, while P4HB with hydrogel barrier was used primarily in intraperitoneal positions due to its anti‐adhesive barrier. Data collection and analyses were coordinated and managed at Erasmus University Medical Center in Rotterdam, the Netherlands.

### 2.2 Participants

Eligible patients were adults (≥ 18 years) with a documented diagnosis of IH who underwent surgical repair using P4HB biosynthetic mesh. Patients were included only if they had previously consented to data collection within national or institutional registries and had follow‐up data available. Exclusion criteria included prophylactic mesh placement (i.e., without hernia), mesh placement for stoma site closure, or diastasis recti repair.

Patients were classified according to the Ventral Hernia Working Group (VHWG) grading system to stratify risk related to contamination and comorbidities [13]. For analytical purposes, patients were grouped as VHWG grade I–II versus grade III–IV.

### 2.3 Data Collection

Clinical data were extracted from registry entries and medical records, including demographic information, comorbidities, operative details, and postoperative outcomes. Collected variables included age, sex, body mass index (BMI), smoking status, diabetes, immunosuppressive conditions, American‐society for Anesthesiology (ASA) score, number of previous hernia repairs, mesh size and location, mesh fixation method, and presence of surgical drainage.

Outcomes were collected at early postoperative follow‐up and long‐term follow‐up (≥ 12 months). The primary outcome was the incidence of long‐term mesh‐related complications occurring two to five years postoperatively. These included:

- Chronic pain
- Mesh explantation (due to infection or pain)
- Enterocutaneous fistula
- Late‐onset mesh infection
- Any reintervention related to mesh complications

Secondary outcomes included:

- Hernia recurrence was defined as a palpable or radiologically confirmed fascial defect in the area of previous hernia repair. A recurrent incisional hernia is defined as an incisional hernia that has previously been surgically repaired.
- Short‐term postoperative complications, including SSI and SSO.
- Clavien‐Dindo classification of postoperative complications (with ≥ Grade III considered major) [14].

SSO was defined as the occurrence of one or more of the following: cellulitis, SSI, seroma, hematoma, wound dehiscence, sinus, enterocutaneous fistula, or infected mesh. SSI was classified as superficial or deep in accordance with CDC guidelines and by DeBord et al. [15].

### 2.4 Statistical Analysis

All data were anonymized and compiled into a centralized database. Descriptive statistics were reported as means with standard deviations or medians with interquartile ranges, depending on distribution. Categorical variables were reported as frequencies and percentages. Between‐group comparisons were made using the chi‐squared or Fisher's exact test for categorical variables and Student's *t*‐test or Mann–Whitney *U* test for continuous variables, as appropriate. Normality was assessed via Q‐Q plots.

A Kaplan–Meier survival analysis was performed to evaluate time to hernia recurrence, and a Cox proportional hazards model was used to identify potential risk factors for recurrence, provided the proportionality assumption was met. Univariable analyses on BMI, type of P4HB mesh, ASA score, location of the mesh (onlay, intra‐peritoneal, retro‐rectus), recurrent hernia (including the number of recurrences), and VHWG score as the predictors were conducted. Variables with a *p*‐value < 0.10 in univariable analyses were included in the multivariable model. Further description on the statistical analyses can be found in our published protocol paper [12].

### 2.5 Handling of Missing Data

In case of missing data, we applied multiple imputation using chained equations (MICE), accounting for clustering by center. Imputed datasets were analyzed separately, and estimates were pooled using Rubin's rules.

### 2.6 Ethics and Data Management

The study used de‐identified data from existing registries, all of which obtained informed consent from patients. Data transfer and storage complied with the EU General Data Protection Regulation (GDPR). Final data analysis and storage were conducted at Erasmus MC using encrypted, password‐protected systems.

## 3 Results

### 3.1 Demographics

A total of 790 patients were included across 7 European registries, with a median follow‐up of 38 months (IQR 36–48). Long‐term follow‐up, defined as longer than 24 months, was available for 448 patients (56.7%). Patient demographics are presented in Table 1 for the different registries across VHWG classes and for the patients with and without follow‐up longer than 1 year. The mean age at surgery was 59 years (SD 14), with little variation across groups, whereas BMI was lowest in VHWG I (24 kg/m^2^) and highest in VHWG II and III (30 kg/m^2^). Male sex was slightly more common overall (53%), although the sex distribution was balanced across subgroups. Smoking status did not differ between grades with 26% in VHWG II, 16% in VHWG III, and 23% in VHWG IV being active smokers. Similarly, diabetes prevalence was equal in VHWG II (32%), VHWG III (26%) and VHWG IV (31%). ASA classification varied, with higher proportions of grade II and III patients across all groups, though VHWG II had the highest proportion of ASA III (54%). The incisional hernia being a recurrent hernia was common overall (62%), particularly in VHWG II (70%).

**TABLE 1:** Baseline characteristics of the patients stratified by follow‐up time and VHWG groups.

Characteristic	Overall follow‐up	Follow‐up < 1 year	Follow‐up > 1 year	Overall VHWG groups	VHWG^b^ I	VHWG^b^ II	VHWG^b^ III	VHWG^b^ IV
	*N* = 790^a^	*N* = 342^a^	*N* = 448^a^	*N* = 645^a^	*N* = 34^a^	*N* = 216^a^	*N* = 286^a^	*N* = 109^a^
Country								
France	370 (47%)	249 (73%)	121 (27%)	356 (55%)	13 (38%)	97 (45%)	197 (69%)	49 (45%)
Germany	115 (15%)	14 (4.1%)	101 (23%)	0 (0.0%)	0 (0%)	0 (0%)	0 (0.0%)	0 (0.0%)
Greece	55 (7.0%)	17 (5.0%)	38 (8.5%)	39 (6.0%)	2 (6%)	9 (4%)	22 (8%)	6 (6%)
Italy	32 (4.1%)	6 (1.8%)	26 (5.8%)	32 (5.0%)	8 (24%)	23 (11%)	1 (0%)	0 (0%)
Spain	218 (28%)	56 (16%)	162 (36%)	218 (34%)	11 (32%)	87 (40%)	66 (23%)	54 (50%)
Sex								
Female	365 (47%)	145 (44%)	220 (50%)	303 (47%)	18 (53%)	83 (39%)	143 (50%)	59 (54%)
Male	404 (53%)	188 (56%)	216 (50%)	337 (53%)	16 (47%)	128 (61%)	143 (50%)	50 (46%)
Age at surgery^c^	59 (14)	61 (14)	57 (14)	59 (14)	55 (16)	58 (13)	60 (14)	59 (15)
BMI^d^	29.6 (6.5)	29.8 (6.2)	29.5 (6.8)	29.8 (6.5)	24.3 (4.7)	30.3 (6.3)	30.1 (6.7)	29.8 (5.8)
Smoking								
Never smoked	471 (60%)	218 (64%)	253 (56%)	360 (56%)	32 (94%)	147 (68%)	225 (79%)	79 (72%)
Stopped smoking	36 (4.6%)	25 (7.3%)	11 (2.5%)	31 (0.5%)	2 (6%)	12 (6%)	14 (5%)	5 (5%)
Active smoker	254 (35%)	99 (29%)	78 (41%)	254 (39%)	0 (0%)	57 (26%)	47 (16%)	25 (23%)
Diabetes								
No	478 (61%)	233 (68%)	245 (55%)	356 (55%)	34 (100%)	147 (68%)	213 (74%)	75 (69%)
Yes	312 (39%)	109 (32%)	203 (45%)	289 (45%)	0 (0%)	69 (32%)	73 (26%)	34 (31%)
Cirrhosis								
No	772 (98%)	335 (98%)	437 (98%)	628 (97%)	34 (100%)	202 (94%)	285 (100%)	107 (98%)
Yes	18 (2%)	7 (2%)	11 (2%)	17 (3%)	0 (0%)	14 (6%)	1 (0%)	2 (12%)
Immunosuppression use								
No	634 (80%)	308 (90%)	326 (73%)	455 (71%)	34 (100%)	189 (88%)	257 (90%)	95 (87%)
Yes	156 (20%)	34 (10%)	122 (27%)	190 (29%)	0 (0%)	27 (13%)	29 (10%)	14 (13%)
Anticoagulation								
No	531 (70%)	265 (79%)	266 (63%)	403 (66%)	24 (92%)	161 (83%)	240 (84%)	96 (88%)
Yes	259 (30%)	77 (21%)	182 (37%)	210 (34%)	2 (8%)	32 (17%)	45 (16%)	13 (12%)
COPD								
No	558 (71%)	279 (82%)	279 (62%)	431 (67%)	34 (100%)	181 (84%)	238 (83%)	92 (84%)
Yes	232 (29%)	63 (18%)	169 (38%)	214 (33%)	0 (0%)	35 (16%)	48 (17%)	17 (16%)
ASA^e^								
I	149 (19%)	67 (20%)	82 (18%)	72 (11%)	5 (15%)	23 (11%)	40 (14%)	4 (4%)
II	283 (36%)	136 (40%)	147 (33%)	269 (42%)	16 (47%)	75 (35%)	120 (42%)	58 (53%)
III	342 (43%)	130 (38%)	212 (47%)	293 (45%)	13 (38%)	116 (54%)	121 (43%)	43 (39%)
IV/V	16 (2%)	9 (3.0%)	7 (2%)	11 (2%)	0 (0%)	2 (1%)	5 (2%)	4 (4%)
Recurrent hernia								
No	330 (42%)	159 (46%)	171 (38%)	243 (38%)	13 (38%)	65 (30%)	121 (42%)	44 (40%)
Yes	460 (58%)	183 (54%)	277 (62%)	402 (62%)	21 (62%)	151 (70%)	165 (58%)	65 (60%)

^a^ *n* (%); Mean (SD).

^b^ Ventral Hernia Working Group grade.

^c^ In years.

^d^ Body‐mass index in (kg/m^2^).

^e^ American Society for Anesthesiology score.

[Correction added on 21 Mar 2026, after first online publication: Table values are updated.]

### 3.2 Short‐Term Complications

**FIGURE 1:**
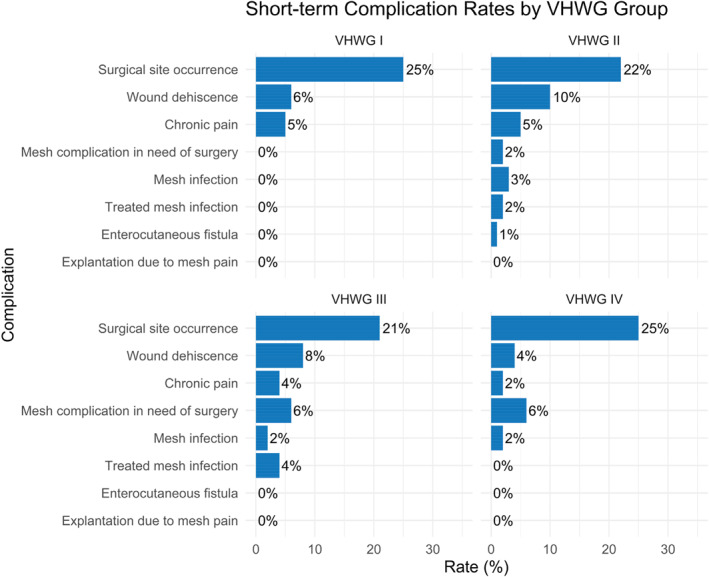
Postoperative complications within 30 Days.

The majority of complications occur during the first 6 months. Within 30‐day the complication rate differed between the VHWG groups with primarily the mesh complications in need of reoperation and the mesh infection. The plots of the cumulative incidence complications can be seen in Figure 1.

### 3.3 Long‐Term Complications

**FIGURE 2:**
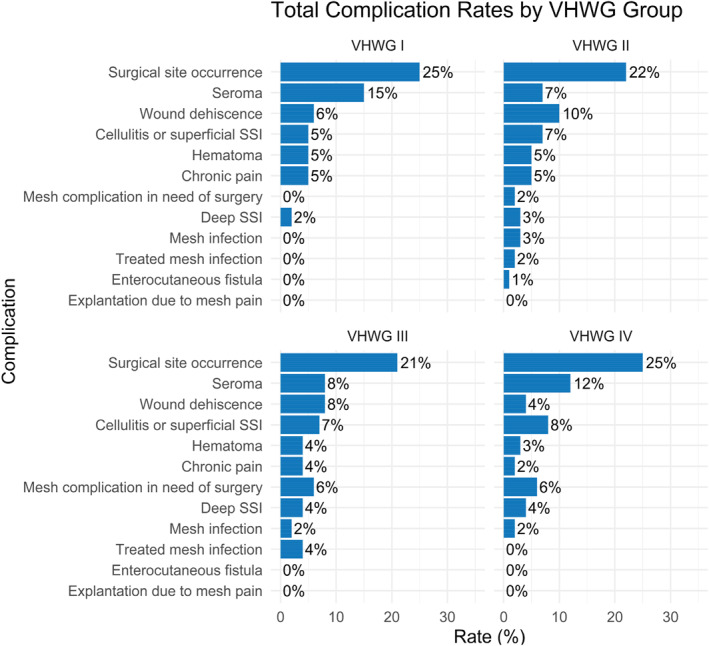
Total long‐term complications.

At long‐term follow‐up the complication rate differed between the VHWG groups with primarily the mesh complications in need of reoperation, the deep SSI and the superficial SSI increasing. The plots of the incidence of the complications can be seen in Figure 2.

Patients with primary incisional hernias showed comparatively low complication rates, with SSO occurring in 17.3%, superficial and deep SSI in 5.3% and 3.9%, and seroma and hematoma in 6.4% and 3.6%, respectively. Mesh‐related morbidity was limited, with mesh infection in 2.2% and chronic pain in 3.1%. In patients undergoing repair for a first recurrence, overall morbidity was higher, including an SSO rate of 19.8%, superficial SSI of 9.0%, seroma of 7.5%, and wound dehiscence of 8.2%, while mesh infection remained low (2.2%) but chronic pain increased to 5.2%. Patients with two or more recurrences demonstrated the highest complication burden, with SSO rising to 29.9%, seroma to 14.5%, and hematoma to 6.9%, although no mesh infections or enterocutaneous fistulas were observed; chronic pain occurred in 4.8%.

### 3.4 Hernia Recurrence

**FIGURE 3:**
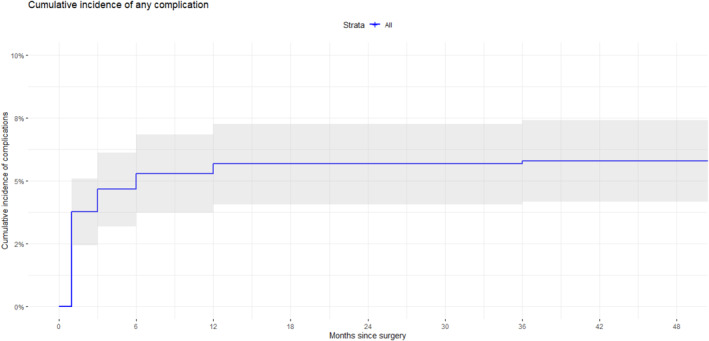
Overall surgical complication rate evolution during the follow‐up.

**FIGURE 4:**
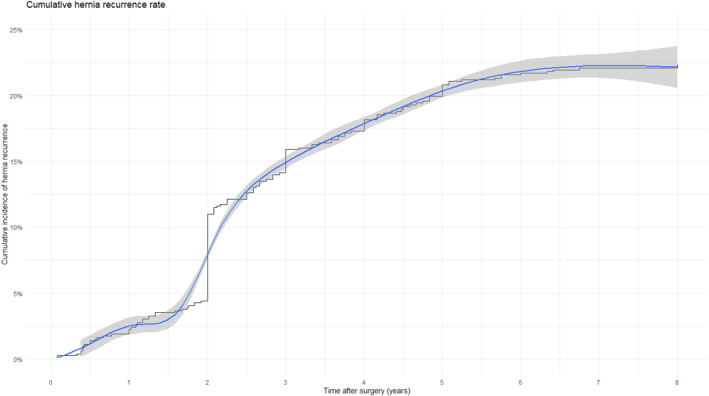
Cumulative hernia recurrence rate.

The overall recurrence rate of incisional hernias in the cohort was 22.3% (Figure 3). Mean time till censoring was 35 months (95% CI 33.3–36.3) with a median of 32 months (IQR 24–58). Of the recurrences 66.4% were diagnosed by CT scan, 14.0% by ultrasound, and 19.6% by physical examination. The cumulative incidence curve shows an increase in hernia recurrence after the resorption of the Phasix mesh with a incidence of recurrence that stays constant afterward (Figures 3 and 4). Stratified by VHWG score, recurrences occurred in 19%, 21%, 22%, and 23% in VHWG I through IV, respectively.

The proportional hazards assumption of the cox‐model was not violated and therefore the model was used (Figure S1). The Cox proportional hazards model identified several variables that were significantly associated with the risk of recurrence. The VHWG classification was a strong predictor of recurrence. Compared to patients classified as VHWG I, those in VHWG III had a significantly increased hazard of recurrence (HR = 2.55; 95% CI: 1.32–5.01; *p* = 0.007), as did patients in VHWG IV (HR = 2.49; 95% CI: 1.23–5.09; *p* = 0.014). Although the hazard ratio was elevated in VHWG II (HR = 1.74; 95% CI: 0.83–3.66), this did not reach statistical significance (*p* = 0.145). Obesity (BMI > 30) was also associated with a significantly increased risk of hernia recurrence (HR = 1.41; 95% CI: 1.02–1.94; *p* = 0.037). Regarding mesh placement location, recurrence rates in the IPOM group, onlay mesh group, and sublay mesh group were 39%, 16%, and 16%, respectively. Patients who underwent an intraperitoneal onlay mesh (IPOM) repair had a significantly higher recurrence risk compared to the onlay mesh group (HR = 2.72; 95% CI: 1.29–5.74; *p* = 0.008). In contrast, the sublay technique showed no significant difference in recurrence risk in comparison to the onlay mesh group (HR = 1.01; 95% CI: 0.51–1.98; *p* = 0.985). Finally, the use of a Phasix ST mesh was at the limit of significance in terms of recurrence compared to standard Phasix mesh (HR = 1.51; 95% CI: 1.00–2.29; *p* = 0.05). No statistically significant associations were observed for the ASA classification. The model performance was good, with a concordance index of 0.709 (SE = 0.024).

### 3.5 Relationship to Other Complications

The predictors that were used for the univariate regression models for the other complications were the ASA status, VHWG class, BMI score, the location of the mesh, and the type of P4HB used. Univariate models showed significant relationships with various factors (*p* < 0.1). These factors were included in the multivariate analyses (Tables 2 and 3 and Supporting Information S1: Tables A–E). Regarding seroma formation, no predictors were statistically significant, but sublay mesh placement showed a borderline association with increased odds of seroma (OR = 4.14, 95% CI: 0.96–17.93, *p* = 0.057).

**TABLE 2:** The effect of the factors in multivariate analyses on wound dehiscence.

Factor	Estimate	Std. error	Statistic	Odds ratio	95% Confidence interval	*p* value
ASA^a^						
1 or 2	Ref	Ref	Ref	Ref	Ref	Ref
3	0.77	0.58	1.33	2.15	(0.70, 6.66)	0.183
4 or 5	0.25	0.58	0.43	1.29	(0.41, 4.04)	0.665
Mesh location						
IPOM^b^	Ref	Ref	Ref	Ref	Ref	Ref
Sublay	1.02	0.47	2.17	2.78	(1.10, 7.00)	0.030
Onlay	1.98	0.57	3.45	7.23	(2.35, 22.20)	0.000
Recurrent hernia						
First	Ref	Ref	Ref	Ref	Ref	Ref
Second	0.05	0.37	0.13	1.05	(0.51, 2.17)	0.897
Third or more	0.47	0.38	1.22	1.59	(0.76, 3.35)	0.221
VHWG^c^						
I	Ref	Ref	Ref	Ref	Ref	Ref
II	−0.69	0.46	−1.50	0.44	(0.18, 1.07)	0.135
III	−0.37	0.40	−0.93	0.69	(0.32, 1.15)	0.355
IV	0.14	0.48	0.29	1.15	(0.45, 2.92)	0.775

^a^ American Society for Anesthesiology.

^b^ Intra‐peritoneal mesh placement.

^c^ Ventral‐hernia working group grade.

**TABLE 3:** The effect of the factors in multivariate analyses on chronic pain.

Factor	Estimate	Std. error	Statistic	Odds ratio	95% Confidence interval	*p* value
ASA score^a^						
1 or 2	Ref	Ref	Ref	Ref	Ref	Ref
3	1.88	1.04	1.81	6.54	(0.85, 50.12)	0.071
4 or 5	2.37	1.03	2.31	10.68	(1.43, 79.71)	0.021
BMI^b^	0.04	0.02	2.15	1.04	(1, 1.09)	0.031
Phasix mesh type						
Phasix	Ref	Ref	Ref	Ref	Ref	Ref
Phasix ST	−0.57	0.34	−1.66	0.57	(0.29, 1.11)	0.096

^a^ American Society for Anesthesiology.

^b^ Body mass index.

Onlay mesh placement compared to IPOM was significantly associated with increased odds of wound dehiscence (OR = 7.23, 95% CI 2.35–22.0, *p* < 0.001), as was sublay placement (OR = 2.78, 95% CI 1.10–7.99, *p* = 0.030). Higher ASA physical status classes were significantly associated with increased odds of mesh infection, with ASA III patients having an OR of 6.26 (95% CI 2.18–18.0, *p* = 0.0006) and ASA IV or V patients an OR of 7.93 (95% CI 2.79–22.5, *p* < 0.0001) and also with a higher odds of chronic pain (ASA IV or V OR of 10.68 (95% CI 1.43–79.71)). Additionally, mesh placement onlay (OR = 2.86, 95% CI 1.17–6.99, *p* = 0.021) and sublay (OR = 2.20, 95% CI 1.17–4.13, *p* = 0.014) were associated with increased risk of mesh infection compared to IPOM.

In contrast, onlay and sublay mesh placements were significantly associated with reduced odds of the need of treatment for a mesh infection compared to the IPOM technique, with ORs of 0.09 (95% CI 0.01–0.81, *p* = 0.032) and 0.13 (95% CI 0.04–0.44, *p* = 0.001), respectively. For chronic pain, ASA classes IV or V were significantly associated with increased risk (OR = 10.7, 95% CI 1.43–79.7, *p* = 0.021). BMI was positively associated with chronic pain risk (OR = 1.04 per unit, 95% CI 1.00–1.09, *p* = 0.031).

## 4 Discussion

This study represents one of the most comprehensive long‐term evaluations of IH repair using P4HB mesh, encompassing a large, heterogeneous cohort drawn from multiple registries. With a median follow‐up duration of 38 months and detailed data on late complications, the findings provide meaningful insight into the durability and complication profile associated with fully resorbable synthetic mesh. An overall hernia recurrence rate of 22.6% was observed, which aligns with previous reports on resorbable meshes [3]. However, direct comparison is limited by variability in patient selection, operative technique, and duration of follow‐up across studies. Notably, the cumulative incidence curve indicated a peak in recurrence following the expected resorption period of the P4HB mesh, after which recurrence rates plateaued [4, 8]. This temporal association underscores the critical period during which tissue remodeling must compensate for mesh degradation, highlighting the importance of robust host integration. The Ventral Hernia Working Group classification emerged as a significant predictor of recurrence, with patients in Classes III and IV exhibiting over twice the recurrence hazard compared to those in Class I [3]. These findings emphasize the need for judicious patient selection when employing resorbable mesh in higher‐risk populations. Obesity also independently predicted recurrence, consistent with prior literature citing elevated intra‐abdominal pressure and impaired wound healing as contributing mechanisms [1, 3].

Mesh placement technique significantly influenced recurrence risk. IPOM placement was associated with doubled recurrence rate compared to the onlay approach, while the sublay technique did not demonstrate a statistically significant difference in comparison to the onlay technique [2, 10, 16]. These findings suggest a potential biomechanical or integration‐related advantage of extraperitoneal placements in the context of resorbable meshes.

Complication rates varied markedly across VHWG classifications, with most complications occurring within a year of treatment. Patients classified as VHWG III and IV experienced higher rates of mesh‐related complications, including superficial and deep surgical site infections (SSI) and reoperations. Multivariate regression analysis identified several independent predictors of specific complications. Higher ASA classification was significantly associated with increased risk of mesh infection, chronic pain, and other adverse outcomes, reflecting the broader physiological vulnerability of patients with systemic comorbidities. ASA Classes III and IV/V conferred a six‐to eightfold increased risk of mesh infection. These findings suggest the need for enhanced postoperative surveillance and potentially more conservative surgical planning in high‐ASA patients.

In terms of technique, both onlay and sublay placements were associated with significantly higher odds of mesh infection relative to IPOM placement. Paradoxically, these extraperitoneal placements were also linked with lower odds of requiring surgical intervention for infection, suggesting that superficial infections may be more amenable to conservative management. In contrast, IPOM‐related infections may necessitate mesh explantation due to intra‐abdominal involvement. Wound dehiscence was more prevalent among patients receiving onlay or sublay mesh placements, likely reflecting greater tension and tissue manipulation in these approaches. Chronic pain, a burdensome long‐term complication, was significantly associated with higher ASA classification and increasing BMI, potentially reflecting mechanisms involving systemic inflammation, nerve irritation, and delayed healing. Notably, patients in ASA Class IV experienced more than a tenfold increased risk of chronic pain, underscoring the importance of perioperative optimization and, where appropriate, consideration of non‐surgical management strategies.

Mesh selection remains a pivotal consideration in hernia repair, particularly in high‐risk or contaminated settings. While synthetic non‐resorbable meshes, such as those based on polypropylene or polyester, remain the standard for clean cases due to their mechanical strength and low recurrence rates, they are associated with notable long‐term complications including chronic pain, infection, and fistula formation [2, 10, 17]. Recurrence rates with synthetic mesh in clean VHWG I–II populations are generally reported at 10%–15%, with mesh infection rates as high as 5%–10% in contaminated fields [2, 18, 19]. Resorbable meshes such as P4HB were developed to mitigate these complications in high‐risk or contaminated environments [10]. P4HB has demonstrated favorable integration and low long‐term infection rates, owing to complete resorption within 12–18 months [10]. However, the recurrence rate observed in this study (22.6%) suggests that, in some patients, mesh resorption may outpace the development of sufficient tissue reinforcement.

If the true VHWG I–II recurrence rate with resorbable meshes is in the 15%–20% range (matching many reported rates for permanent synthetic prostheses in clean cases), then slowly resorbable prostheses could be a viable alternative in selected patients with low‐risk of complications. However, definitive conclusions require prospectively collected, adequately imaged long‐term follow‐up and randomized comparisons in clean settings to quantify the trade‐off between recurrence and the well‐documented long‐term complications of permanent synthetics. Until these trials are completed, we could propose the following recommendations: (I) VHWG I–II (clean, relatively low‐risk cases): permanent synthetic mesh remains the gold standard, (II) VHWG III (contaminated fields): biosynthetic meshes such as P4HB may be appropriate, particularly when infection risk is elevated; however, careful follow‐up is essential due to increased recurrence risk, (III) VHWG IV (gross contamination): The benefit of using a slowly resorbable prosthesis to prevent recurrence seems acceptable given the relatively low risk of long‐term complications.

This study has several limitations. Because this study relies on retrospective registry data from multiple countries, reporting bias is an inherent limitation. Variability in documentation practices across centers may influence the accuracy of complication and recurrence reporting. Additionally, incisional hernia recurrence was diagnosed using different modalities (including CT scan, ultrasound, and clinical examination) which may introduce diagnostic heterogeneity and affect recurrence estimates. Lastly, mesh selection and surgical technique were not randomized and may reflect institutional practices or surgeon expertise.

No controlled comparisons could be made with synthetic resorbable and non‐resorbable meshes in our study. Additionally, a major limitation of this study is that only 57% of patients had follow up beyond 24 months, which restricts the strength of any conclusions regarding long term outcomes. Follow up duration is influenced by censoring after recurrence or reoperation, meaning that a substantial proportion of patients do not have follow‐up beyond 24 months. Consequently, stratified comparisons between patients with shorter and longer follow up could not reliably be performed.

Furthermore, no quality of life assessment was conducted with specific abdominal wall questionnaires such as the Hernia‐Related Quality‐of‐Life Survey (HerQles) or Abdominal Hernia‐Q (AHQ) questionnaires which would give valuable insights into the effects of biosynthetic meshes [20, 21]. Finally, functional outcomes and patient‐reported quality‐of‐life metrics were not comprehensively captured, limiting insights into broader postoperative recovery.

## 5 Conclusion

This study highlights the complex interplay of factors influencing recurrence and postoperative complications following IH repair with resorbable mesh. VHWG classification, ASA status, BMI, and mesh placement technique were all independently associated with clinical outcomes. While resorbable meshes such as P4HB demonstrate favorable safety profiles in contaminated or high‐risk fields, recurrence remains a prominent concern, particularly in patients with advanced VHWG classification. However, the risk/benefit balance between complications and long‐term recurrence could favor P4HB prostheses. These findings support an individualized, risk‐stratified approach to mesh selection and surgical planning in abdominal wall reconstruction.

## Author Contributions

**Rudolf van den Berg:** conceptualization, methodology, writing – original draft, formal analysis, resources, investigation, software, project administration, visualization. **Marie Wieser:** conceptualization, methodology, writing – original draft, formal analysis, resources, investigation, software, project administration, visualization. **Manuel López‐Cano:** review and editing. **José Bueno‐Lledó:** review and editing. **Ferdinand Kockerling:** writing – review and editing, investigation, validation, data curation. **Grigorios Chatzimavroudis:** writing – review and editing, investigation, validation, data curation. **Constanza Gonella‐Pacchiotti:** investigation, validation, data curation, writing – review and editing. **Cesare Stabilini:** writing – review and editing, investigation, validation, data curation. **Pablo Ortega‐Deballon:** review and editing. **Benoit Romain:** conceptualization, investigation, writing – original draft, methodology, formal analysis, supervision, resources, data curation.

## Funding

The authors have nothing to report.

## Ethics Statement

The authors have nothing to report.

## Conflicts of Interest

F.K. received travel grants from Dahlhausen and PFM medical and honouraria for lectures from B.D., L.N.J., G.P., M.L.‐C., J.‐F.G., J.B.‐L., G.C., G.S. and Y.R. reported financial relationships with BD, including lectures, educational activities, grants and/or advisory roles. P.O., J.‐F.G., D.M. and Y.R. reported involvement with Cousin Surgery through speaker fees, consultancy or educational honouraria. L.N.J., G.P., M.L.‐C., J.‐F.G. and Y.R. also had ties with Medtronic for similar activities. V.D. and B.R. reported receiving grants from B.D., G.P. and D.M. disclosed financial relationships with Gore. P.O. reported speaker fees from Smith and Nephew, and J.‐F.G. reported participation in a study supported by Intuitive and involvement with Swing THT. All other authors declare no conflicts of interest.

## Supporting information


Supporting Information S1

**Figure S1:** Cox‐regression assumption.

## Data Availability

Data used in the study are available from the authors upon reasonable request.

## Appendix A

### PRS Group (Phasix Research Study Group)

Authors and affiliations

Authors part of PRS Group (Phasix Research Study Group): Giorgio Soliani, Lars N. Jorgensen, Jose Antonio Pereira Rodriguez, Jean‐François Gillion, Vincent Dubuisson, Niki Christou, Yohann Renard, David Moszkowicz, Guillaume Péré, Gregory Baud, Guillaume Passot, Benjamin Barrat, J. Jeekel.

Department of Surgery, S. Anna University Hospital and University of Ferrara, Ferrara, Italy

Digestive Disease Center Bispebjerg Hospital, University of Copenhagen, Copenhagen, Denmark

General and Digestive Surgery Department, Parc de Salut Mar, Hospital del Mar, Barcelona, Spain

Unité de Chirurgie Viscérale et Digestive, Hôpital Privé d'Antony, Antony, France

Department of Digestive and Endocrine Surgery, Bordeaux University Hospital (CHU), Bordeaux, France

Endocrine, General and Digestive Surgery Department, CHU of Limoges, Limoges, France

Department of General Surgery, Reims Champagne‐Ardenne University, Reims, France

Université Paris Cité, Service de Chirurgie Générale et Digestive, DMU ESPRIT—GHU AP‐HP Nord—Université Paris Cité, Colombes, France

Department of Digestive Surgery, Esogastric Bariatric and Endocrinal Surgery Unit, Toulouse‐ Rangueil University Hospital, Toulouse, France

Endocrine, General and Digestive Surgery Department, CHU of Lille, France

Department of Surgical Oncology, Hopital Lyon Sud, Hospices Civils de Lyon, Pierre Bénite, France

Department of Neuroscience, Erasmus University Medical Centre, Rotterdam, the Netherlands
