# Supplementary material for: Long‐Term Outcomes After Slowly Resorbable P4HB Mesh Implantation: A Multicenter Analysis From European Registry
Source: World J Surg. 2026 Mar 20;50(5):1195–205. doi: 10.1002/wjs.70331 (PMC13206534; doi:10.1002/wjs.70331)
Supplement: Supplementary file 2 — Figure S1: Cox‐regression assumption. [file WJS-50-1195-s002.pdf]

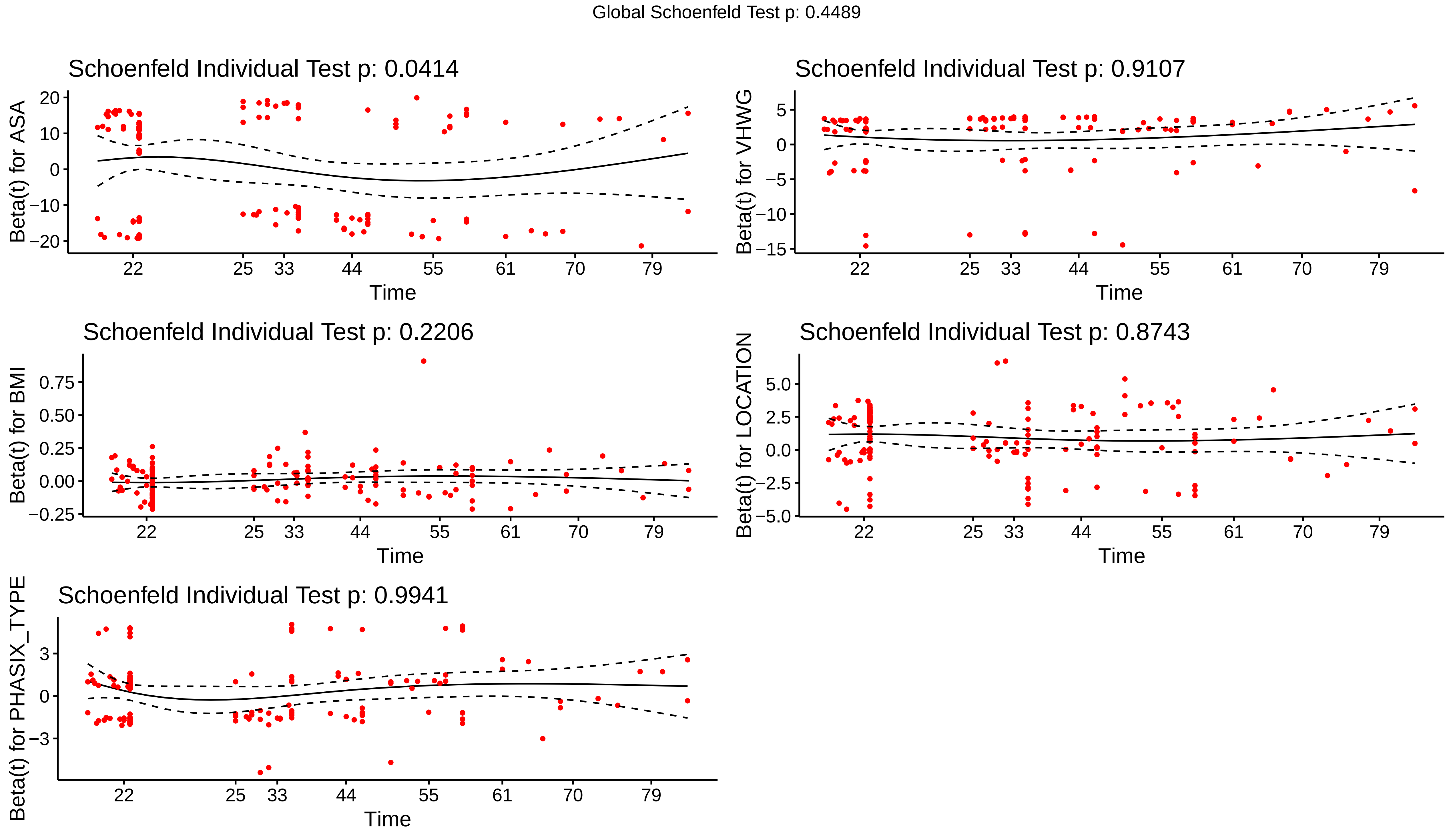

Global Schoenfeld Test p: 0.4489
Schoenfeld Individual Test p: 0.0414
Beta(t) for ASA
Time
Schoenfeld Individual Test p: 0.9107
Beta(t) for VHWG
Time
Schoenfeld Individual Test p: 0.2206
Beta(t) for BMI
Time
Schoenfeld Individual Test p: 0.8743
Beta(t) for LOCATION
Time
Schoenfeld Individual Test p: 0.9941
Beta(t) for PHASIX_TYPE
Time
22 25 33 44 55 61 70 79
